# Effects of Acute Exercise and Training on the Sarcoplasmic Reticulum Ca^2+^ Release and Uptake Rates in Highly Trained Endurance Athletes

**DOI:** 10.3389/fphys.2020.00810

**Published:** 2020-07-07

**Authors:** Kasper Degn Gejl, Erik P. Andersson, Joachim Nielsen, Hans-Christer Holmberg, Niels Ørtenblad

**Affiliations:** ^1^Department of Sports Science and Clinical Biomechanics, University of Southern Denmark, Odense, Denmark; ^2^Swedish Winter Sports Research Centre, Department of Health Sciences, Mid Sweden University, Östersund, Sweden; ^3^Department of Physiology and Pharmacology, Biomedicum C5, Karolinska Institute, Stockholm, Sweden

**Keywords:** sarcoplasmic reticulum, fatigue, Ca^2+^ handling, athletes, exercise, training

## Abstract

Little is presently known about the effects of acute high-intensity exercise or training on release and uptake of Ca^2+^ by the sarcoplasmic reticulum (SR). The aims here were to characterize this regulation in highly trained athletes following (1) repeated bouts of high-intensity exercise and (2) a period of endurance training including high-intensity sessions. Eleven cross-country skiers (25 ± 4 years, 65 ± 4 mL O_2_⋅kg^−1^⋅min^–1^) performed four self-paced sprint time-trials (STT 1-4) lasting ≈ 4 min each (STT 1–4) and separated by 45 min of recovery; while 19 triathletes and road cyclists (25 ± 4 years, 65 ± 5 mL O_2_⋅kg^−1^⋅min^–1^) completed 4 weeks of endurance training in combination with three sessions of high-intensity interval cycling per week. Release (μmol⋅g^–1^ prot⋅min^–1^) and uptake [tau (s)] of Ca^2+^ by SR vesicles isolated from m. *triceps brachii* and m. *vastus lateralis* were determined before and after STT 1 and 4 in the skiers and in m. *vastus lateralis* before and after the 4 weeks of training in the endurance athletes. The Ca^2+^ release rate was reduced by 17–18% in both limbs already after STT 1 (arms: 2.52 ± 0.74 to 2.08 ± 0.60; legs: 2.41 ± 0.45 to 1.98 ± 0.51, *P* < 0.0001) and attenuated further following STT 4 (arms: 2.24 ± 0.67 to 1.95 ± 0.45; legs: 2.13 ± 0.51 to 1.83 ± 0.36, *P* < 0.0001). Also, there was a tendency toward an impairment in the SR Ca^2+^ uptake from pre STT1 to post STT4 in both arms and legs (arms: from 22.0 ± 3.7 s to 25.3 ± 6.0 s; legs: from 22.5 ± 4.7 s to 25.5 ± 7.7 s, *P* = 0.05). Endurance training combined with high-intensity exercise increased the Ca^2+^ release rate by 9% (1.76 ± 0.38 to 1.91 ± 0.44, *P* = 0.009), without altering the Ca^2+^ uptake (29.6 ± 7.0 to 29.1 ± 8.7 s; *P* = 0.98). In conclusion, the Ca^2+^ release and uptake rates by SR in exercising limbs of highly trained athletes declines gradually by repetitive bouts of high-intensity exercise. We also demonstrate, for the first time, that the SR Ca^2+^ release rate can be enhanced by a specific program of training in highly trained athletes, which may have important implications for performance parameters.

## Introduction

The functional capacity of skeletal muscles relies partly on intrinsic metabolic and mechanic properties and by repetitive contractions these may be disrupted leading to a reduction in skeletal muscle function and performance, i.e., fatigue (Allen et al., 2008). In most activation patterns and exercise tasks, a substantial part of the fatigue development is associated with impairments in the sequence of events at the muscle level leading to muscle activation and relaxation denoted the excitation-contraction (E-C) and relaxation coupling. The E-C and relaxation is a strictly coordinated regulation of the free cytosolic Ca^2+^ concentration in order to control the myofiber contraction and relaxation. This regulation is handled through an intracellular membrane-delimited organelle, the sarcoplasmic reticulum (SR). In response to muscle activation by action potential propagation throughout the t-tubules, the SR Ca^2+^ release channel [ryanodine receptor (RyR1)] opens, and Ca^2+^-ions diffuse passively into the cytosol, elevating the cytosolic Ca^2+^ 10 to 20-fold (Ingalls et al., 1999; Bruton et al., 2003) activating the cross-bridge cycling. Concomitantly, Ca^2+^-ions are re-sequestered back to the SR through another SR membrane protein [SR Ca^2^ ATPase (SERCA)], leading to relaxation of the muscle fiber. Studies in intact single fibers of rodent and human skeletal muscle have repeatedly revealed that steps in the E-C coupling involving myofibrillar Ca^2+^ regulation are an important part of the decrease in force production following repeated contractions, with the underlying cellular mechanisms being, (i) an impaired SR Ca^2+^ release, (ii) a reduced myofibrillar Ca^2+^ sensitivity or (iii) a reduced maximal Ca^2+^ activated force production, with the importance of each depending on muscle activation pattern and duration (Allen et al., 2008). Further, these studies have revealed decreases in muscle fiber relaxation rate and rates of myofibrillar Ca^2+^ decline.

While myofibrillar Ca^2+^ levels have mainly been studied in rodent single fiber preparations, SR Ca^2+^ release and uptake rates can be measured directly in SR vesicles from human biopsies. In these studies, deteriorations in both SR vesicle Ca^2+^ release and uptake have repeatedly been reported in response to exercise. A decreased SR vesicle Ca^2+^ release and -uptake has been observed following short-term high-intensity exercise in untrained individuals (Hostrup et al., 2014) and following a single bout of short-term high-intensity knee-extension exercise or endurance exercise in both untrained (Booth et al., 1997; Tupling et al., 2000; Duhamel et al., 2006) and trained individuals (Li et al., 2002; Ørtenblad et al., 2011; Gejl et al., 2014). The few studies in highly trained individuals have shown consistent reductions in the SR vesicle Ca^2+^ release rate following exhaustive endurance exercise, whereas changes in SR Ca^2+^ uptake have been less consistent (Li et al., 2002; Ørtenblad et al., 2011; Gejl et al., 2014). In line with this, extensive fragmentation of the SR Ca^2+^ release channel was observed in recreationally active human subjects following repeated high-intensity exercise, while experiments on elite endurance athletes performing the same exercise showed no channel fragmentation (Place et al., 2015). However, the existing literature in trained humans has primarily focused on changes in SR function with acute endurance exercise, while little is known about changes during repeated high-intensity exercise and recovery.

A systematic change in function and demands posed on skeletal muscle will result in adaptations that increase performance toward the characteristics of the exercise stimulus (Bogdanis, 2012). Accordingly, adaptations that may reduce muscle fatigue during high-intensity exercise depend on the characteristics of the training program, i.e., type, intensity, frequency, and duration. While a salient response to training is increases in e.g., mitochondrial content and function (Granata et al., 2018) the antioxidant capacity (Hellsten et al., 1996; Ørtenblad et al., 1997) and muscle glycogen availability (Gejl et al., 2017a) little is known about effects of training on the SR Ca^2+^ handling properties *per se* in athletes. In untrained individuals, however, the SR Ca^2+^ ATPase activity has been shown to remain unaltered following sprint training (Ørtenblad et al., 2000) and resistance training (Green et al., 1998; Hunter et al., 1999) in some studies, while others have observed reductions following sprint training (Harmer et al., 2014) resistance training (Hunter et al., 1999) and aerobic training (Green et al., 2003). To the best of our knowledge, only one study has investigated the effects of exercise training on the SR Ca^2+^ release rate, and here a robust increase in SR vesicle Ca^2+^ release rate was observed following a 5-week period with sprint training (Ørtenblad et al., 2000). Based on these findings, high-intensity exercise seems necessary to improve the SR Ca^2+^ handling properties and it is highly relevant to investigate whether such adaptations in the SR Ca^2+^ handling are also present following exercise training involving high-intensity exercise in highly trained athletes. Accordingly, an optimization of the SR regulatory abilities may prevent myocellular disturbances in the Ca^2+^ homeostasis during exhaustive exercise.

Here, we examined changes in SR Ca^2+^ uptake and release in arm and leg muscles of highly trained athletes in response to acute exercise and training – i.e., cross-country skiers performing repeated high-intensity exercise and triathletes and road cyclists conducting 4 weeks of endurance training including high-intensity exercise, i.e., both interventions similar to their actual activities. We hypothesized that both acute and repeated high-intensity exercise impair SR Ca^2+^ handling, whereas a period of endurance training including high-intensity exercise improves SR function.

## Materials and Methods

### Study Overview

**FIGURE 1:**
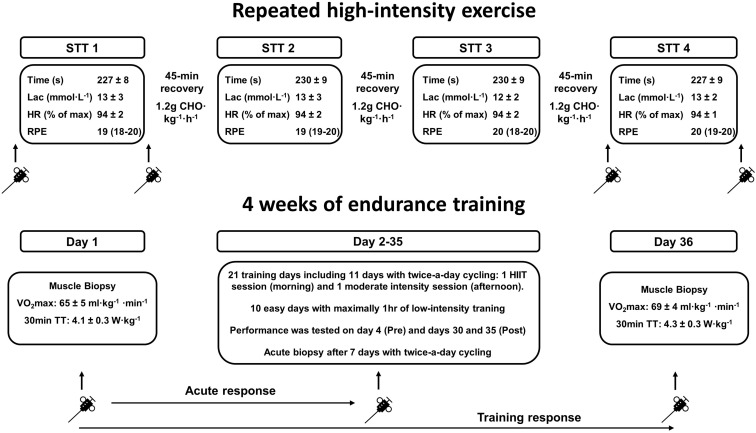
Overview of the study protocol employed to examine the response of calcium handling by the sarcoplasmic reticulum in the muscles of the arms and legs to exercise. The **(upper)** section illustrates the four maximal 1300-m sprint time-trials (STT 1–4), separated by 45-min periods of recovery with intake of carbohydrate (CHO), performed by highly trained cross-country skiers, as well as associated analyzes. Muscle biopsies were obtained before and after the first (STT1) and fourth time-trials (STT4). Information about the time required to complete (time), blood lactate concentration (Lac) immediately after completion, average heart rate (HR) and RPE during each STT have been reported previously [values are mean ± SD, and for RPE as median (range)] (Andersson et al., 2016). The **(lower)** section illustrates the “real-life” training day and subsequent 34-day period of training by highly trained triathletes and road cyclists (described in detail in the section “Materials and Methods”). Information about changes in performance (i.e., VO2max and power output during the 30-min time-trial) following this training period have also been reported previously (Gejl et al., 2017b).

The effects of both acute exercise and training on SR vesicle Ca^2+^ handling was determined in two groups of elite athletes performing exercise resembling “real-life” competition or training (Figure 1 and Table 1). One group of highly trained cross-country skiers performed acute exercise consisting of four bouts of sprint time-trials (STTs) separated by 45 min. of recovery between bouts. The other group consisting of highly trained triathletes and road cyclists carried out both acute exercise and 4 weeks of endurance training including high-intensity exercise.

**TABLE 1:** Subject characteristics.

	Repeated high- intensity exercise	Acute responses to endurance training	Long-term responses to endurance training
**Group**	Cross-country skiers	Triathletes and Cyclists	Triathletes and Cyclists
***n***	11	12	19
**Age** (years)	25 ± 4	24 ± 4	25 ± 5
**Body mass** (kg)	79 ± 6	76 ± 7	74 ± 7
**Height** (cm)	183 ± 9	184 ± 7	182 ± 6
**VO_2max_** (mL⋅min^–1^)	5.1 ± 0.5	5.0 ± 0.6	4.8 ± 0.6
**VO_2max_ (**mL⋅kg^−1^⋅min^–1^)	65 ± 4	66 ± 6	65 ± 5
**MHC Leg** (% I; % IIa)	51 ± 8; 49 ± 8	53 ± 7; 47 ± 7	51 ± 8; 49 ± 8
**MHC Arm** (% I; % IIa)	39 ± 6; 61 ± 6		

*Highly trained cross-country (CC) skiers underwent four bouts of high-intensity exercise with 45 min recovery, while highly trained triathletes and road cyclists performed exercise of moderate- to high intensity for 4 weeks. At different time-points during this period, SR Ca^2 +^ handling of muscles in the arms and legs of the skiers and legs of the endurance athletes was examined. The values presented are means ± SD.*

### Subjects and Ethical Approval

The 11 cross-country skiers who performed STTs were highly trained male competitors in national and/or international sprint and distance races who trained 8–11 h each week during the period of the study (Table 1). The group performing endurance training was composed of 13 highly trained male triathletes and 6 elite male road cyclists. Among the triathletes, six were current members of the Danish National Team competing in international Olympic and sprint distances (the Olympic Games, World Triathlon Series, World Cup and Continental Cup), 7 participated in elite international and national competitions (at Olympic, ½ ironman and ironman distances), while the remaining two competed at a lower level (½ ironman and ironman distances). All 6 cyclists had A-licenses and competed at the elite national level (Table 1).

The participants were fully informed of potential risks associated with the experiments before verbal and written consents were obtained. The study was approved by the local Ethics Committee of Southern Denmark (Project-ID S-20150034) and Regional Ethics Review Board in Umeå, Sweden (#2013-59-31) and the experiments adhered to the standards of the *Declaration of Helsinki*. This study was part of a larger project described in part previously (Gejl et al., 2017b).

### The STTs

The skiers were tested on a treadmill in the laboratory (Mid Sweden University, Östersund, Sweden) on separate days over 3 weeks, with at least 48 h between test days. These tests are described in detail elsewhere (Andersson et al., 2016). Briefly, baseline height and body mass, as well as maximal oxygen uptake (V̇O_2max_) while performing both diagonal and double poling techniques were determined during the first visits, while the penultimate visit involved familiarization with the simulated competition protocol and on the final day the actual testing took place.

The protocol included four STTs, each 1300 m of supra-maximal roller-skiing (∼4 min. in duration), with 45 min. of recovery between successive STT’s (Figure 1). Before undertaking STT 1, the skier warmed-up for 15-20 min., while STT 2-4 were preceded by a 5-min warm-up and STT 1–3 followed by a 5-min cool-down. Between cool-down and warm-up, the skiers rested passively for approximately 28 min.

Each 1300 m STT consisted of three flat sections (1° incline) to be skied employing the double poling technique (DP), with two intermediate uphill sections (7° incline) on which the diagonal stride sub-technique (DS) was utilized. The course was in total, 57% flat (1°), 23% uphill (7°), while the remaining 20% consisted of transitions from 1° to 7° or vice versa. This resulted in an average course incline of 2.8°. Skiers were encouraged to exert a maximal effort during each trial and were aided in doing so by continuous feedback concerning their speed and position on the course provided by a screen in front of the treadmill. The course was designed to simulate international cross-country sprint competitions (except downhill sections for safety reasons) and the total duration of the four STTs and recovery periods was 3 h, i.e., similar to the duration of actual sprint competitions.

Skiing speed, heart rate and V̇O_2_ were monitored continuously during the four STTs, and this data subsequently averaged (Andersson et al., 2016; Gejl et al., 2017a). Room temperature (∼22°C) and humidity (∼56%) were maintained throughout the experiment. The subjects were instructed to refrain from all physical activities of moderate to high intensity during the 48 h prior to testing.

During the 24 h prior to the first STT, each skier consumed three standardized CHO-enriched meals (on average, 18.000 kJ, 55% CHO, 30% fat, and 15% protein) and three snacks (8 g CHO⋅kg^–1^ bw⋅day^–1^), with the last meal being consumed in the laboratory 120 min. before the warm-up for STT1. During each of the 45-min. recovery periods the subjects consumed 1.2 g CHO⋅kg^–1^ bw⋅h^–1^, on average 40 g CHO in the form of a sport drink mixed with water and 30 g as an energy gel, with *ad libitum* intake of water. This CHO intake during the recovery periods was in accordance with the recommendations from the American College of Sports Medicine (Rodriguez et al., 2009).

### Endurance Training

The triathletes and cyclists performed 4 weeks of routine training in combination with high-intensity interval cycling (HIIT). Skeletal muscle SR vesicle Ca^2+^ handling properties were examined at rest both before and after this 4-week intervention, as well as 1h following the moderate-intensity afternoon session on the seventh day of this intervention (Figure 1).

Originally, the study was designed to investigate the effects of periodized glycogen depletion on adaptations to training and for this purpose CHO intake during certain portions of the training period was manipulated. However, and as reported in companion papers, all acute responses (e.g., muscle glycogen, serum hormone levels, blood glucose) and training adaptations (e.g., performance, oxidative enzyme activity, VO_2_max) were not different and similar between the intervention groups despite the CHO manipulation (Gejl et al., 2017b, 2018). This was also the case regarding SR Ca^2+^ handling, which allowed us to combine these two groups for the present analysis.

With the advice of exercise researchers and the Danish national triathlon coach, 4-week training plans were designed for the triathletes and cyclists, taking training history into account. On average, athletes trained 16 [12–20] hours per week and performed three HIIT sessions per week (11 times in total). The HIIT session was performed in the morning and consisted of eight 5-min cycling intervals separated by 2 min of active recovery, followed 7 h later by a moderate intensity cycling session. In connection with each session of HIIT, the first six 5-min intervals were conducted with a target intensity of 85% HR_max_, while the final two consisted of five 15-s maximal sprints designed to recruit type II fibers, separated by 45-s of easy spinning. The afternoon session entailed 2 hrs of moderate cycling with a target intensity of 65% HR_max_.

Both morning and afternoon sessions were carried out on personal bikes by use of turbo trainers (Tacx Bushido Smart T2780, Wassenaar, Netherlands). The three weekly double sessions comprised 30–50% of the total training volume. For the purposes of recovery, 1–2 weekly training days involved a maximum of 45 min of easy training. The remaining training days included two sessions of easy-to-moderate biking for the road cyclists (1.5–3.5 h at 65–75% of HR_max_), or in the case of the triathletes, 2–4 sessions of easy-to-moderate swimming and 4–6 easy-to-moderate runs (65–85% of HR_max_). Thus, in connection with this intervention, which was conducted during the pre-season, both the intensity of exercise during HIIT and the amount of high-intensity exercise were greater than normal for this period, whereas the total overall volume of training was maintained and similar to normal training during this period.

On the 11 days including two bikes sessions, all meals were provided to the subjects, while on the remaining days subjects were instructed to ingest CHO-enriched meals in the morning, for lunch and for dinner. To ensure dietary conformity, 3-day dietary recordings were collected both prior to and during the training period. A few athletes were asked to increase energy intake if their total intake of energy or CHO was less than recommended [1.8 PAL (0.0669bw + 2.28) MJ⋅day^–1^ or 5g CHO kg^−1^⋅day^–1^]. Detailed information about training and dietary intake during the intervention has been described elsewhere (Gejl et al., 2017a).

### Analytical Procedures

#### Muscle Biopsies

Using 5 mm Bergström needles, muscle biopsies of 100–150 mg were obtained from the *m. triceps brachii* and *m. vastus lateralis* in skiers and the *m. vastus lateralis* in the triathletes and cyclists. The procedure for the extraction of muscle tissue has been described in detail elsewhere (Gejl et al., 2017b). Muscle biopsies were obtained randomly from the right and left limb. In the skiers, biopsies were obtained before and after both STT 1 and 4. The first biopsy was extracted 35 min before STT 1 while the remaining biopsies were obtained 10–12 min after STT1 and 10–12 min before and after STT 4.

In the triathletes and cyclists, biopsies were obtained before (Pre) and after (Post) the 4 weeks of training as well as acutely (Acute) following the 7th day including both HIIT and moderate-intensity exercise. The pre- and post-biopsies were obtained in 19 subjects 1h after a standard meal and at identical time-points within each subject. The subjects received a standard diet for the last 24 h prior to the biopsy extraction (5 g CHO ⋅ kg bm^−1^⋅d^–1^ with a total energy intake of 34.8 kcal ⋅ kg bm^–1^). The final day with twice-a-day cycling was completed 36 h before extraction of the post-biopsy and athletes refrained from exercise during this recovery period. The acute biopsy was obtained in 12 subjects 1 h after completing the moderate-intensity afternoon session.

In both the skiers and the group of triathletes and cyclists, the muscle samples were placed on a filter paper on an ice-cooled ∼0°C petri dish and divided into several specimens. One part of the biopsy was immediately frozen in liquid nitrogen and stored at −80°C for subsequent analysis of glycogen content. Another part was manually homogenized with a potter-elvehjem glass-glass homogenizer (Kontes Glass Industry, Vineland, NJ, United States) for determination of SR vesicle Ca^2+^ release- and uptake and myosin heavy chain (MHC) distribution.

#### SR Vesicle Ca^2+^ Uptake and Release

**FIGURE 2:**
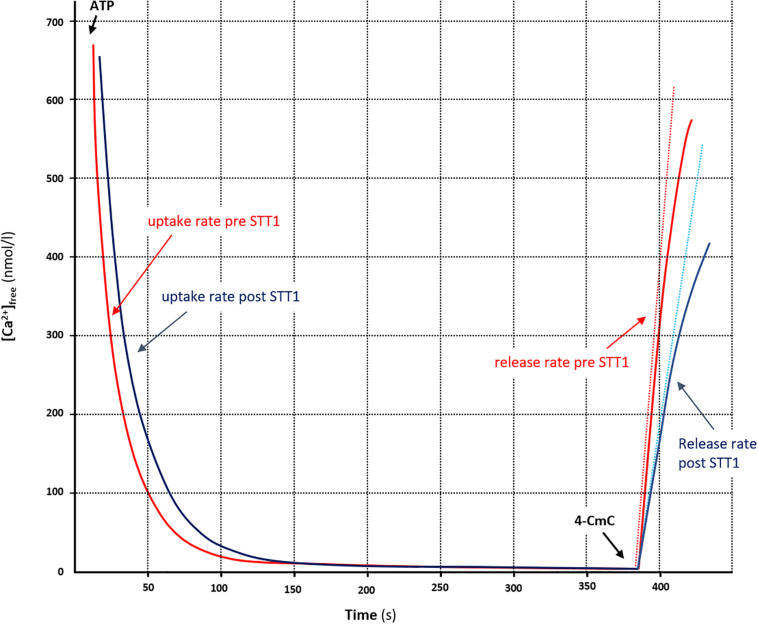
Measurement of parameters associated with SR vesicle Ca^2+^ uptake and -release from representative examples of [Ca^2+^] traces in arm muscle, pre (STT1, red trace) and post (STT 2, blue trace) high-intensity exercise (∼4 min. in duration). The free [Ca^2+^] was determined fluorometrically and the SR vesicle Ca^2+^ uptake was initiated by adding ATP (5 mM). When [Ca^2+^] reached a plateau, Ca^2+^ release was initiated by adding 4-CmC (5 mM). Curve fitting of Ca^2+^ uptake was performed with data points between a free [Ca^2+^] of 700 nM and the free [Ca^2+^] 20 s prior to initiating Ca^2+^ release. The time (τ) to reach 63% of the SR vesicle uptake (i.e., the initial free [Ca^2+^] minus nadir-Ca^2+^) was calculated as 1/b from the equation; [Ca^2+^]_free_ = ae-bt +c (see section “Materials and Methods”). SR Ca^2+^ release rate was obtained by mathematically fitting the data points during the first 30 s of release to the equation: *y* = x(1 – e-y(t-z), back-extrapolate to nadir-Ca^2+^ and then the rate of Ca^2+^ release was determined as the derivative of the initial release (dotted lines).

The fluorescent dye technique was used to determine Ca^2+^ uptake and release rates in SR vesicles and it has been described in detail elsewhere (Ørtenblad et al., 2011; Gejl et al., 2014). Free [Ca^2+^] was determined by the fluorescent Ca^2+^ indicator Indo-1 (1 μM) (20 Hz, Ratiomaster RCM; Photon Technology International, Brunswick, NJ, United States). SR vesicle Ca^2+^ uptake was initiated by adding 2 mM ATP to a final concentration of 5 mM and Ca^2+^ uptake was recorded for 3 min, before [Ca^2+^] reached a plateau. The SR Ca^2+^ uptake rate was defined as the time for free [Ca^2+^] to decrease by 63%. Upon measurements of Ca^2+^ uptake, the SR Ca^2+^ ATPase was blocked with cyclopiazonic acid before SR vesicle Ca^2+^ release was initiated by addition of 4-chloro-M-Cresol (4-CmC) (5 mM). Raw-data for [Ca^2+^] were mathematically fitted using mono-exponential equations as previously described (Curve Fitting Toolbox version 1.1.1; The MathWorks, Natick, MA, United States) (Ørtenblad et al., 2011; Gejl et al., 2014). A representative example of measurements before and after STT 1 in the cross-country skiers is illustrated in Figure 2. Values obtained for SR Ca^2+^ release rates are relative and expressed as arbitrary units; Ca^2 +^ ⋅g protein^−1^⋅min^–1^. Assays of Ca^2+^ uptake and release were performed in triplicates (a few in duplicates due to limited tissue homogenate). Protein content in the muscle homogenate was measured in triplicates using a standard kit (Pierce BCA protein reagent no. 23225).

#### Muscle Glycogen and MHC Isoform

Muscle glycogen content was determined spectrophotometrically (Beckman DU 650) (Passonneau and Lowry, 1993; Gejl et al., 2014). Freeze-dried muscle tissue (1.5 mg) was boiled in 0.5 ml 1 M HCL for 150 min before it was quickly cooled, whirl-mixed and centrifuged at 3500 *g* for 10 min at 4°C. Forty μL of boiled muscle sample and 1 ml of reagent solution containing Tris-buffer (1M), distilled water, ATP (100 mM), MgCl_2_ (1M), NADP^+^ (100 mM) and G-6-PDH were mixed before the process was initiated by adding 10 μL of diluted hexokinase. Absorbance was recorded for 60 min before the glycogen content was calculated. Muscle glycogen was expressed as mmol⋅kg dw^–1^.

Myosin heavy chain (MHC) composition was determined from homogenate using gel electrophoresis as previously described (Betto et al., 1986) and modified for humans (Ortenblad et al., 2000). Briefly, muscle homogenate (80 μL) was mixed with 200 μL of sample-buffer (10% glycerol, 5% 2-mercaptoethanol and 2.3% SDS, 62.5 mM Tris and 0.2% bromophenolblue at pH 6.8.), boiled in water at 100°C for 3 min and loaded (10–40 μL) on a SDS-PAGE gel [6% polyacrylmide (100:1 acrylmid : bis-acrylmid), 30% glycerol, 67.5 mM tris-base, 0.4% SDS, and 0.1 M glycine]. Gels were run at 80 V for at least 42 h at 4°C and MHC bands made visible by staining with Coomassie. The gels were scanned (Lino-scan 1400 scanner, Heidelberg, Germany) and MHC bands quantified densitometrically (Phoretix 1D, non-linear, Newcastle, United Kingdom) as an average of the three loaded protein amounts. MHC II was identified with Western blot using monoclonal antibody (Sigma M 4276) with the protocol Xcell IITM (Invitrogen, Carlsbad, CA, United States).

### Statistical Analysis

All interactions or main effects were tested using a linear mixed-effects model, with time (and limb for the skiers) as fixed effects and individual subjects as random effect. Assumptions on heteroscedasticity and normal distribution were evaluated by inspecting the distribution of residuals and a standardized normal probability plot, respectively. Values are expressed as means ± SD and sample sizes are illustrated in the figure legends. Pearson’s correlation analysis was used to analyze potential associations between variables. Analyzes demonstrating *P* ≤ 0.05 were considered significant. All statistical analyzes were performed using Stata, version 16 (StataCorp LP, College Station, TX, United States). We have previously demonstrated a significant negative effect of exercise on the SR Ca^2+^ release rate (Gejl et al., 2014). Based on this data, with a mean reduction of −0.50 μmol ⋅ g prot^–1^ ⋅ min^–1^ and a mean SD of 0.35, a minimum sample size of 5 subjects was calculated as needed to attain a power of 0.80.

## Results

### SR Ca^2+^ Handling in Skiers Performing Repeated High-Intensity Exercise

**FIGURE 3:**
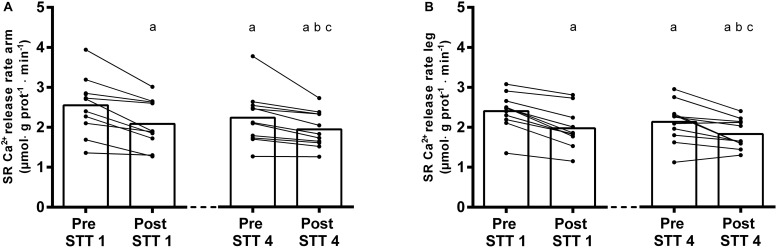
SR Ca^2+^ release rates in arms **(A)** and legs **(B)** of elite cross-country skiers before (Pre) and after (Post) the first (STT 1, *n* = 10) and the fourth (STT 4, *n* = 11) bout of 1300-m high-intensity skiing. Each bout was interspersed with 45 min of recovery including carbohydrate intake. Both individual changes and mean values are shown; a different from Pre STT1, b different from Post STT1, c different from Pre STT4. See text for exact *p*-values.

**FIGURE 4:**
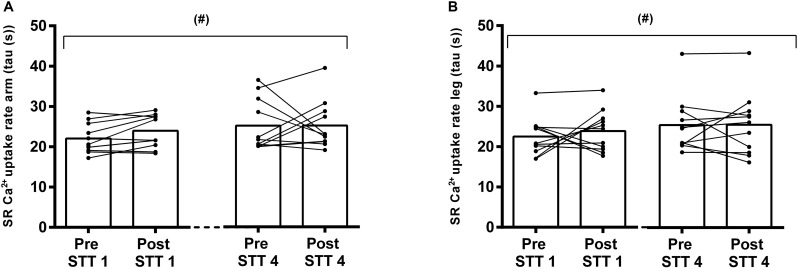
SR Ca^2+^ uptake measured as the time for free [Ca^2+^] to decrease by 63% in arms **(A)** and legs **(B)** of elite cross country skiers before (Pre) and after (Post) the first (STT 1, *n* = 10) and the fourth (STT 4, *n* = 11) bouts of 1300 m high-intensity skiing. Each bout was interspersed with 45 min of recovery including carbohydrate intake. Both individual changes and mean values are shown; (#) tendency to a main effect of time, *P* = 0.053.

The simulated sprint cross-country skiing competition, consisting of 4 × 4 min of high-intensity exercise interspersed with 45 min recovery periods, lead to significant fluctuations in the SR Ca^2+^ handling in both arms and legs (Figures 3, 4).

#### SR Ca^2+^ Release Rate

The exposure to a single bout of 4 min high-intensity exercise (i.e., STT 1) induced a significant reduction in the SR Ca^2+^ release rate (arms: from 2.52 ± 0.74 to 2.08 ± 0.60 μmol ⋅ g prot^–1^ ⋅ min^–1^; legs: from 2.41 ± 0.45 to 1.98 ± 0.51 μmol ⋅ g prot^–1^ ⋅ min^–1^, Pre vs. Post: *P* < 0.0001 in both cases) (Figures 3A,B). After two additional bouts of high-intensity exercise (STT 2 and 3) and the intermediate 45 min periods of recovery, these rates were still depressed (arms: 2.24 ± 0.67 μmol ⋅ g prot^–1^ ⋅ min^–1^; legs: 2.13 ± 0.51 μmol ⋅ g prot^–1^ ⋅ min^–1^, *P* < 0.0001).

Moreover, during the fourth bout of high-intensity exercise (i.e., STT 4), the SR Ca^2+^ release rate were further reduced by 13–14% (arms: 1.95 ± 0.45 μmol ⋅ g prot^–1^ ⋅ min^–1^; legs: 1.83 ± 0.36 μmol ⋅ g prot^–1^ ⋅ min^–1^, *P* < 0.0001 in both cases) (Figures 3A,B). Although there was no limb × time interaction in this context (*P* = 0.99), the overall Ca^2+^ release rate was slightly higher in the arms than in the legs (*P* = 0.04).

#### SR Ca^2+^ Uptake

The Ca^2+^ uptake data from the skiers showed an unequal variance of the residuals across the time points. However, the tendency for a main time effect (*P* = 0.053) was confirmed after an inverse transformation of the data (*P* = 0.098), where equal variance was achieved (Figures 4A,B). In this context, there was no limb × time interaction (*P* = 0.99), and no differences between limbs (*P* = 0.99) (Figures 4A,B). Accordingly, there was a gradual numerical decline in the SR Ca^2+^ uptake from pre STT1 to post STT4 in both arms and legs (arms: from 22.0 ± 3.7 s to 25.3 ± 6.0 s; legs: from 22.5 ± 4.7 s to 25.5 ± 7.7 s).

### “Real-Life” Endurance Exercise and SR Ca^2+^ Handling

**FIGURE 5:**
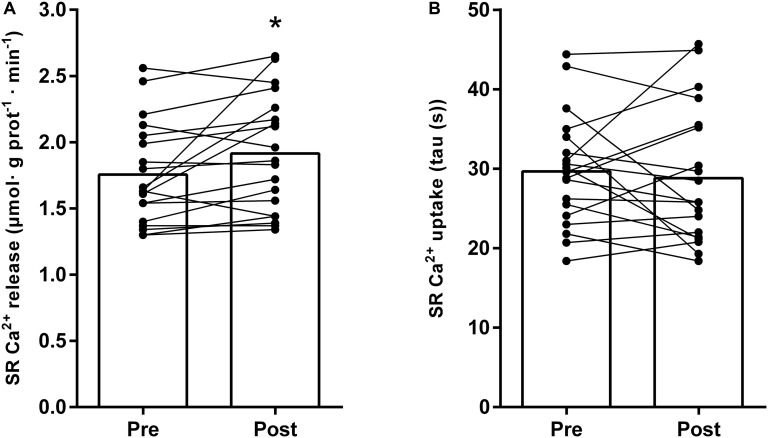
Changes in SR vesicle Ca^2+^ release rate **(A)** and SR Ca^2+^ uptake **(B)**, measured as the time for free [Ca^2+^] to decrease by 63%, during 4 weeks of endurance training in highly trained endurance athletes (*n* = 19). The training period included a mixture of low-, moderate-, and high-intensity exercise – *see* section “Materials and Methods” for details. Both individual and mean changes are shown. ^∗^ different from pre, *P* = 0.009.

Like the repeated high-intensity exercise, the conduction of a strenuous “real-life” training day in highly trained triathletes and cyclists lead to a significant depression of the SR Ca^2+^ release rate (from 1.82 ± 0.31 μmol ⋅ g prot^–1^ ⋅ min^–1^ to 1.40 ± 0.23 μmol ⋅ g prot^–1^ ⋅ min^–1^, *P* < 0.0001), whereas the SR Ca^2+^ uptake was unaffected (26.6 ± 6.9 s to 31.6 ± 6.4 s, *P* = 0.12). Following the entire 4-week intervention, the SR Ca^2+^ release rate was elevated by 9% (from 1.76 ± 0.38 to 1.91 ± 0.44 μmol ⋅ g prot^–1^ ⋅ min^–1^, *P* = 0.009) (Figure 5A), still without any change in uptake (29.6 ± 7.0 versus 29.1 ± 8.7 s; *P* = 0.98) (Figure 5B).

### Relationships Between SR Ca^2+^ Handling and Fiber Type Distribution and Endurance Performance

None of the groups demonstrated any association between the fiber type distribution (i.e., the relative MHC II content) and the SR Ca^2+^ release rate (cross-country skiers: *r*^2^ = 0.01, *P* = 0.80; triathletes and cyclists: *r*^2^ = 0.01, *P* = 0.61), or uptake rate (cross-country skiers: *r*^2^ = −0.16, *P* = 0.26; triathletes and cyclists: *r*^2^ = 0.03, *P* = 0.21). Nor was endurance performance (i.e., the time required by the skiers to complete STT1 or the power output by the endurance athletes during the 30-min time-trial, Figure 1) correlated to either Ca^2+^ release (cross-country skiers: *r*^2^ = 0.16; triathletes and cyclists: *r*^2^ = 0.01) or uptake (cross-country skiers: *r*^2^ = 0.25; triathletes and cyclists: *r*^2^ = 0.16) (Gejl et al., 2017b).

## Discussion

The present findings provide novel insights into the effects of acute and repeated high-intensity exercise, as well as a period of endurance training, on SR Ca^2+^ handling in the arm and leg muscles of highly trained athletes. While existing studies in endurance athletes have shown reduced SR Ca^2+^ release rates following single bouts of maximal leg-extension exercise (Li et al., 2002) and endurance exercise (Ørtenblad et al., 2011; Gejl et al., 2014) the present findings further extend this knowledge by demonstrating that as little as 4 min of whole-body high-intensity exercise attenuates the rate of SR Ca^2+^ release in both the arms and legs, with a further reduction when exercise is repeated. Moreover, short-term recovery (i.e., 45 min) appeared to partly reverse this effect. In contrast, 4 weeks of endurance training including high-intensity exercise by highly trained endurance athletes increased the SR Ca^2+^ release rate.

SR Ca^2+^ uptake was less affected by acute high-intensity exercise, showing only a non-significant reduction in both arms and legs after one bout, but then a significant decline in both sets of limbs following 3 and 4 bouts. In the case of 4 weeks of endurance training including high-intensity exercise, the SR Ca^2+^ uptake rate was not changed either acutely after a single day of demanding training or at rest following the training period.

### Effects of Acute Exercise on SR Ca^2+^ Handling

The maximal rate by which the SR releases Ca^2+^ can only be determined in SR vesicles and are carried out *in vitro* under apparently optimal conditions and demonstrate that once the SR is removed from the intracellular environment and not under normal voltage sensor control, changes in function persist. Thus, changes in the release rate can solely be explained by a modulation of the RyR1 opening probability and/or changes in the content of RyR1 in the muscle fibers. The underlying mechanism(s) explaining the present observations of a decreased SR vesicle Ca^2+^ release rate by acute exercise has been related to either oxidative modulations of the RYR1 and a fragmentation of the channel. Place and colleagues recently demonstrated that 6 × 30 s of “all-out” cycling exercise in untrained individuals led to a ROS-induced fragmentation of RyR1 and consequently an increased SR Ca^2+^ leak with reductions in SR Ca^2+^ release and power output (Place et al., 2015). Still, in elite endurance athletes the same exercise protocol was not associated with a RyR1 fragmentation, despite the power output being reduced (Place et al., 2015). Accordingly, the authors explained the structural maintenance of RyR1 in trained individuals by an increased ROS defense and a reduced ROS production during exercise (Place et al., 2015) and it was suggested that reductions in myofibrillar Ca^2+^ sensitivity could explain the reduction in performance (Westerblad and Allen, 2011). The present results support the absence of a RyR1 fragmentation by acute exposure to high-intensity exercise in trained individuals since the reduction in the SR Ca^2+^ release rate was partly reversed during recovery (Figure 3). However, as reported in a companion study (Gejl et al., 2016) the plasma redox status was disturbed during the repeated high-intensity exercise while the myofibrillar Ca^2+^ sensitivity was improved, possibly as a result of exercise-induced oxidation of the contractile apparatus. Since performance was maintained throughout the series of high-intensity exercise bouts, it is likely that the reductions in SR Ca^2+^ release were, at least partly, compensated for by increases in Ca^2+^ sensitivity (Andersson et al., 2016; Gejl et al., 2017a). Thus, it cannot be excluded that reductions in SR Ca^2+^ release rates were, at least partly, due to ROS-mediated RyR1 modifications. Previously, muscle glycogen depletion has been associated with impairments in SR Ca^2+^ handling (Gejl et al., 2017b) however, in the present studies muscle glycogen was not critically depleted during exercise, and no associations were observed between muscle glycogen availability and SR Ca^2+^ release rate acutely after exercise in any of the groups (*r*^2^ = 0.00–0.29, *P* = 0.09–0.98). We have previously suggested that muscle glycogen depletion negatively affects the SR Ca^2+^ release when below 250–300 mmol ⋅ kg dw^–1^, but in the present studies post-exercise glycogen levels were not significantly below this level (i.e., cross-country skiers: 290 mmol ⋅ kg dw^–1^; endurance athletes:430 mmol ⋅ kg dw^–1^) (Gejl et al., 2017a, 2018).

Concerning the acute effects of exhaustive exercise on SR Ca^2+^ uptake in highly trained athletes, the existing findings are inconsistent by showing impairments in the legs following maximal leg extension exercise (Li et al., 2002) and 1 h of cross-country skiing (Ørtenblad et al., 2011) and maintenance of Ca^2+^ uptake in the legs following 4 h of cycling exercise (Gejl et al., 2014) and the arms following 1 h of cross-country skiing (Ørtenblad et al., 2011). The present findings extend this knowledge by showing that SR Ca^2+^ uptake is unaffected by 4 min of maximal whole-body exercise, whereas the four bouts of high-intensity exercise tended to exert a negative effect. Thus, the Ca^2+^ uptake seems relatively stable unless the muscle is exposed to a certain amount of high-intensity exercise. By investigating SR vesicles, the strong tendency to impairments observed with the repeated high-intensity exercise must be explained by modifications of the SR Ca^2+^-ATPase itself or endogenous regulators of this enzyme. Apart from glycogen, which was not associated with SR Ca^2+^-uptake following exercise in the present studies (*r*^2^ = 0.00 – 0.15, *P* = 0.21–0.83), it is possible that the SR Ca^2+^-ATPase activity was adversely affected by structural modifications of its nucleotide binding site (i.e., oxidation or nitrosylation) as a result of the ROS production during exercise (Klebl et al., 1998; Matsunaga et al., 2003; Tupling, 2004). In addition, high ADP concentrations or low local ATP concentrations in proximity to the SR Ca^2+^-ATPase have been shown to adversely affect pump function *in vitro*, but the presence of such local changes *in vivo* is unknown (Tupling, 2004).

### Effects of Exercise Training on SR Ca^2+^ Handling

Acute deteriorations in SR function, and particularly the SR Ca^2+^ release rate, has repeatedly been demonstrated as an important mechanism underlying the development of muscle fatigue (Li et al., 2002; Duhamel et al., 2006; Gejl et al., 2014; Place et al., 2015) and accordingly, improvements in the SR Ca^2+^ release rate with training could serve as a preventive mechanism, especially during high-intensity exercise. Interestingly, we here demonstrate that the exposure to routine endurance training, with superimposed high-intensity exercise, increased the SR Ca^2+^ release rate by 10% in already trained road cyclists and triathletes. To the best of our knowledge, only one study from our lab has elucidated the effect of high-intensity training on SR function in humans. In this study, a 9% increase in SR vesicle Ca^2+^ release rate was observed following 5 weeks of sprint training in untrained individuals (Ørtenblad et al., 2000). Furthermore, the data indicated that the 5-week sprint training induced an increase in the SR Ca^2+^ release rate, due to an enhanced SR content within the fiber. Altogether, these findings suggest that the muscle SR Ca^2+^ release rate increase in response to high-intensity exercise training, supporting the idea of an adaptation in SR function to counteract muscle fatigue and thereby improving athlete performance. We extracted the post-biopsy 36 h following the last training session, and since it has previously been shown that the RyR1 channel is not deteriorated 24 h following HIIT in endurance trained athletes, we assume that this training session did not affect our results (Place et al., 2015). However, if RyR1 was affected by the last training session, we may have slightly underestimated the positive effect of training.

Since MHC II fibers contain more SR and demonstrate higher SR Ca^2+^ release rates in comparison to MHC I fibers, a change in the fiber type composition could explain alterations in SR function (Rüegg, 1986). However, the fiber type distribution remained unchanged during the training period and, accordingly, this cannot explain the improved SR function in the present study (MHC I: 50 ± 9% to 51 ± 8%; MHC II: 50 ± 9% to 49 ± 8%). Based on previous findings from our lab, an enhanced Ca^2+^ release rate could instead be explained by an increase in the total number of RyR1 secondary to an increase in the total amount of SR per fiber (Ørtenblad et al., 2000). Interestingly, a pharmacologically induced increase in SR Ca^2+^ release rate, by β2-adrenergic stimulation, is associated with an increase in contractile force in non-fatigued muscle in trained men (Hostrup et al., 2014). Thus, increases in the SR Ca^2+^ release rate *per se* may improve muscle contractility as also demonstrated by the present and previous training effects.

Only a few previous studies have examined the SR Ca^2+^ uptake rate following a period of training and only resistance training in elderly women has previously been shown to increase rates of Ca^2+^ uptake, whereas resistance training (Hunter et al., 1999) as well as 5–7 weeks with sprint-training in young individuals (Ørtenblad et al., 2000; Harmer et al., 2014) does not seem induce changes in SR Ca^2+^ uptake. Since we did not observe any significant change following the 4 weeks of endurance training, the present findings are in agreement with previous findings in young untrained individuals. By contrast, 10 weeks of prolonged aerobic training has been shown to induce reductions in the SR Ca^2+^ uptake (Green et al., 2003). Although not measured in the present study, previous studies have reported that intensified training does not affect SR Ca^2+^-ATPase content and -activity, and generally mechanisms related to the SR Ca^2+^ uptake seems less responsive to training in comparison to the SR Ca^2+^ release (Madsen et al., 1994; Green et al., 1998; Ørtenblad et al., 2000).

### Implications of Changes in SR Function for Performance

As presented in a companion study, the repeated periods of high-intensity exercise were not associated with the development of fatigue since the time to complete the first and fourth bout of exercise was identical (STT1: 227 ± 8 s; STT4: 227 ± 9 s) (Gejl et al., 2017a). Interestingly, the performance was maintained despite the marked reductions in SR Ca^2+^ release and -uptake rates, which questions previous proposals of SR Ca^2+^ release rate as being an important mechanism underlying the development of muscle fatigue during exercise (Westerblad and Allen, 1991; Gejl et al., 2014; Place et al., 2015). As mentioned previously, reductions in SR Ca^2+^ release *per se* may compromise muscle function, but since other important steps in the E-C coupling may be improved (e.g., Ca^2+^ sensitivity) (Gejl et al., 2016; Lamboley et al., 2020) a net negative change in muscle function may not appear. Also, the exercise intensity during the repeated bouts of exercise as well as the endurance training day may have been too low to challenge the SR Ca^2+^ handling capacity and it cannot be excluded that the observed reductions in SR Ca^2+^ release rate would compromise performance at even higher exercise intensities or longer durations.

According to the concept of symmorphosis, structures are developed to match the functional capacity of the system, and no single parameter has unnecessary excess capacity (Weibel et al., 1991). Here, we demonstrate a significant increase in the SR Ca^2+^ release rate by 4 weeks of endurance training including 11 high-intensity training sessions. An effect that, according to the concept of symmorphosis, is likely to enhance the maximal exercise capacity of the muscle. This may be particularly important in sports involving intermittent periods of high-intensity exercise and, accordingly, we have previously shown that the ability to perform maximal sprint exercise was reduced following 4 h of moderate intensity exercise concomitantly with a 15% reduction in SR Ca^2+^ release (Gejl et al., 2014).

### Methodological Considerations

Importantly, SR function can only be analyzed *in vitro* and this reductionistic approach cannot account for interactions between different myocellular sites. Therefore, as changes in SR function with acute exercise and training were evident when it was removed from the *in vivo* conditions, it is important to consider the physiological relevance of these changes for whole muscle function. Using this method, it is herein an important assumption that the SR vesicle used for the *in vitro* measurements of SR function is functional apparent in *in vivo* muscles. First, the measures on SR vesicles represent functional measures of the Ca^2+^ uptake and release rates irrespective of possible changes present in the *in vivo* muscle, and changes in the SR function will reflect inherent changes in the SERCA pump and RyR channel, respectively (i.e., redox modifications or fragmentation of the RyR-channel). Second, these changes will be present in the athlete’s muscle and conceivably affect muscle function, although it is not possible to estimate further modifications present in the *in vivo* milieu. Widening the present study to include an investigation of SR protein modifications could possibly have provided an explanation for the observed functional changes. However, the purpose of the present study was to investigate if exercise, that has previously been shown to induce mechanistic alterations in rodents and humans, ultimately affects SR on the functional level.

We have previously observed that SR function remained unchanged in the non-training control group of a 5-week training study (Ørtenblad et al., 2000). Thus, due to the difficulties associated with recruiting elite athletes to invasive studies involving muscle biopsies, and since the inclusion of a control group would comprise a 4-week period without training, which we assumed would not appeal to this specific group, we decided to conduct the project without a control group. Importantly, the included athletes had been training and competing on a high level for at least 2 years and since only the training approach was manipulated during the intervention period, we strongly believe that the observed changes were a result of this.

In conclusion, handling of Ca^2+^ by the SR in the arms and legs deteriorates following both a single bout and multiple bouts of high-intensity cross-country skiing by highly trained skiers. Moreover, we demonstrate that the rate of SR Ca^2+^ release in elite athletes can be enhanced by a specific program of training, which may have important implications for performance.

## Data Availability Statement

The data that support the findings of this study are available from the corresponding author upon reasonable request.

## Ethics Statement

The studies involving human participants were reviewed and approved by the Ethics Committee of Southern Denmark (Project-ID S-20150034) Regional Ethics Review Board in Umeå, Sweden (#2013-59-31). The patients/participants provided their written informed consent to participate in this study.

## Author Contributions

KG, EA, NØ, and H-CH were responsible for the conception or design of the study. KG, NØ, and JN were responsible for acquisition, analysis, or interpretation of data. KG, EA, NØ, H-CH, and JN were responsible for drafting the manuscript or revising it critically for important intellectual content. All the authors have approved the final version of the manuscript. All persons designated as authors qualify for authorship.

## Conflict of Interest

The authors declare that the research was conducted in the absence of any commercial or financial relationships that could be construed as a potential conflict of interest.
